# Meeting Abstract

**DOI:** 10.1002/2211-5463.13954

**Published:** 2025-01-06

## Education Workshops

### AI in bioscience: unleashing potential in teaching and assessment

#### David Smith^1^

##### ^1^ *Sheffield* *Hallam University, UK*

As the integration of artificial intelligence (AI) into various scientific disciplines continues to accelerate, there is a growing need for bioscience educators to harness the power of AI to enhance teaching, research and assessment. The impetus for this workshop stemmed from the recognition that many bioscience academics are either unaware of or under‐equipped to implement generative AI tools in their practices. This workshop was designed to address this gap by providing targeted training in the use of AI, specifically tailored to the bioscience context.

This workshop aimed to empower bioscience academics with the knowledge and skills necessary to integrate AI into their teaching and research methodologies. The objectives were:

- To introduce participants to generative AI tools and their potential applications in bioscience.
- To develop participants' skills in prompt engineering and advanced data analysis, enabling them to guide AI tools towards producing relevant and accurate outputs.
- To explore innovative strategies for incorporating AI into teaching and assessment, enhancing student engagement and learning outcomes.

The workshop featured a blend of theoretical instruction and practical, hands‐on activities. Video tutorials and practical prompts were provided through our AI Forge website*, constructed alongside Dr Nigel Francis. Participants were introduced to the fundamentals of large language models (LLMs), natural language processing (NLP) and the principles of generative AI. Emphasis was placed on understanding how these technologies work and the ethical considerations. A significant portion of the workshop was dedicated to prompt engineering, where participants learned how to craft effective prompts to elicit precise and contextually appropriate responses. Additionally, the workshop provided practical strategies for using AI in teaching and assessment, including personalising student feedback and creating more engaging learning experiences.

At the workshop's conclusion, participants gained a better understanding of AI's potential and limitations in bioscience education. Additionally, they developed an awareness of the ethical responsibilities related to AI use, particularly in upholding academic integrity and ensuring fair access to these powerful tools.

*https://sites.google.com/my.shu.ac.uk/theaiforge/home.

### Incorporating molecular visualisation into the teaching and learning practice: 3D visualisation, structure databases, structure prediction by AI

#### Angel Herráez^1^

##### ^1^ *University* *of Alcalá, Spain*

Experimental determination of macromolecular structure has a long history, though the techniques involved make it stay away from the general community of instructors and students. We perceive a need to facilitate the use of this freely available wealth of information, for easier and richer analysis of biomolecules, interactions and basis for their function, hence overcoming the limitations of classical 2D resources such as textbooks and diagrams.

This was a hands‐on workshop introducing participants into ways of merging interactive, 3D molecular structures both in the teaching routines and in student activities. The main aim was to get acquainted with tools and to explore profitably the information available in repositories, without becoming specialists or going into elaborate procedures.

The following main topics were covered:

- Software to display and interact with the structure of molecules in 3D; we mostly focused on Jmol and JSmol, versatile tools as well as accessible without cost.
- Opportunities offered by the Proteopedia platform to present, share and collaborate in authoring media‐rich educational material.
- Knowledge of the databases where structural information may be obtained: Protein Data Bank, PubChem…
- Introduction to the services of protein structure prediction using artificial intelligence (AI) algorithms (AlphaFold Database, ESM Metagenomic Atlas).

The workshop was attended by 11 participants from eight countries, from Portugal to Malaysia, who practiced the search and retrieval of molecular structure data in the databases, and customised display of features.

They also learned basic use of Proteopedia to embed interactive 3D structures among the textual and graphic content of a webpage. Each participant worked in a personal temporary space, and some of them signed up to work both on their permanent space and in community pages.

Finally, simple methods were explored to obtain predicted 3D structure of proteins in easily accessible AI platforms, as a supplement for those lacking experimental data.

Hopefully, participants will start to include some of these tools in their teaching and spread the word among colleagues on how to easily use them to enrich the education process of their students.

### How do we make group work assignments meaningful and engaging?

#### Nigel Francis^1^, Stephen Rutherford^1^

##### ^1^ *Cardiff* *University, UK*

One of the most sought‐after graduate attributes cited by employers is the ability to work effectively in a team environment. However, group‐based activities, especially group‐based assessments, are typically disliked by students in Higher Education. Group‐based assessments are fraught with challenges and often result in significant problems which need to be resolved and remediated thereby limiting learning gain. How then, can we design group‐based assessments which offer a positive and reaffirming educational experience for students? The key is to focus on preparing students for the group activities by supporting them in teamwork skills and understanding. Proper support and scaffolding of activities can make group‐based assessment meaningful, and provide experiences aligned to the situations our students will face post‐graduation.

This workshop focused on practical ways of designing group tasks to engage students and to empower them as effective team workers. The workshop highlighted the potential challenges and barriers in group assessments, and how best to address these. Participants shared experiences of the challenges they had faced group work assessments, and effective approaches for overcoming these issues. Four key factors for effective group assessments were discussed: (a) clarifying the pedagogic rationale for using group work as the assessment task, the choice of the task itself, and the design of the anticipated group activities; (b) the importance of preparing students for effective team working, including highlighting ways in which teams function and potential roles for individuals within a team. Supporting students to assign roles to team members and set deadlines for deliverables; (c) the monitoring and ongoing support of student groups during the task, through light‐touch personalised monitoring, reflective record‐keeping and rapid identification of problems (with early interventions to solve these); (d) the fair and equitable allocation of marks relative to effort and engagement by group members, and the provision of formative feedback.

The workshop surfaced ideas for the development of team‐based assessments, which are not only effective but also potentially even enjoyable for students undertaking them.

## FEBS Education and Training Academy Workshops

### Bridging science and teaching: active learning strategies for molecular life scientists

#### Manuel João Costa^1^, Ali Burak Özkaya^2^

##### ^1^ *Universidade* *do Minho, Portugal*, ^2^ *Izmir* *University of Economics, Türkiye*

Considering the growing need for effective teaching approaches in molecular life sciences, this workshop addressed the gap between scientific research and teaching practices. The workshop supported molecular science educators in redesigning a course embedding feasible strategies to foster active learning, and enhance student engagement and learning. By guiding educators through the planning of the course redesign, the workshop aimed to:

- Introduce the principles and benefits of active learning.
- Demonstrate effective teaching and assessment techniques in biochemistry and molecular biology.
- Equipe educators with practical tools to implement active learning in their classrooms.

The workshop included three 2‐h sessions, each designed to explore evidence‐based approaches to improving student engagement and understanding in biochemistry and molecular biology. The first session introduced active learning principles and examined the evidence to support the effectiveness of active learning. The second session focused on effective teaching techniques, including interactive lectures, group work and problem‐based learning. The third and final session covered assessment strategies aligned with active learning, including formative assessments and feedback mechanisms.

By the end of the workshop, participants had gained (a) an understanding of the benefits and principles of active learning, (b) practical experience with various teaching techniques tailored to molecular life sciences, (c) knowledge of effective assessment strategies to enhance student learning and (d) confidence in implementing these strategies to create a more engaging and effective learning environment.

### Maximising mentorship impact: empower your PhD supervisory skills

#### Robert A. Harris^1^, Jerka Dumić^2^

##### ^1^ *Karolinska* *Institute, Sweden*, ^2^ *University* *of Zagreb, Croatia*

The purpose of the workshop was to enhance the understanding of participants of the responsibilities of being a supervisor of PhD students and to provide hands‐on experience of aspects of doctoral supervision that are generally considered more challenging—feedback and conflicts.

The workshop encompassed three 2‐h sessions, each designed to focus on supervisor training within the field of biomedical sciences—(a) The professional responsibilities of a supervisor, (b) conflict prevention and conflict management, and (c) the giving and receiving of feedback. These were discussed in breakout and whole group formats in an interactive and storytelling format.

The workshop participants had different degrees of PhD supervisor experience, and this contributed greatly to the peer‐learning concept of the workshop through individual storytelling and sharing of best practices. The varied format of the workshop, including short presentations by workshop leaders, role play of specific supervisor‐PhD student interactions, as well as individual, breakout group and whole group reflections created a mix of learning opportunities that catered to everyone's preference.

### Building career‐ready scientists: a practical guide to assessing, integrating and reflecting on transferable skills in science education

#### Luciane V. Mello^1^, Damjana Kastelic^2^

##### ^1^ *University* *of Liverpool, UK*, ^2^ *Centre* *for Genomic Regulation (CRG), Spain*

A longstanding challenge for educators in higher education is preparing students for their post‐graduation careers. While a solid theoretical foundation is essential, students must also be capable of connecting and applying their knowledge in various contexts valuable to employers. The use of reflection as a learning tool in education is not new but remains underexplored in science education and the workplace. There is a need to foster self‐reflection when transferable skills are embedded and assessed in the curriculum. Additionally, educators should help students find solutions independently, promoting ownership and problem‐solving skills—key to producing career‐ready scientists.

This 3‐day workshop focused on skill development, divided into two main parts: student‐focused and educator development. Participants ranged from postgraduate students to heads of departments, creating a fruitful environment for discussion. The workshop centred on collaborative design through group discussions, facilitating the sharing of knowledge and experiences.

Day one explored different teaching approaches to foster students' skills development and mechanisms to embed them in the curriculum. Day two focused on active listening and coaching models, with concrete tools and techniques for educators to empower their students in problem‐solving skills. Self‐reflection was the basis for all discussions. Day three was dedicated to designing individual practical guides to integrate reflection in activities involving students' transferable skills development and mentoring/coaching.

